# Association between hypertension and deep vein thrombosis after orthopedic surgery: a meta-analysis

**DOI:** 10.1186/s40001-016-0207-z

**Published:** 2016-03-22

**Authors:** Lei Huang, Jie Li, Yong Jiang

**Affiliations:** Department of Orthopedics, The First Affiliated Hospital of Dalian Medical University, No 5, Longbin road, Development Zone, Dalian, 116600 China

**Keywords:** Meta-analysis, Hypertension, Deep vein thrombosis

## Abstract

**Background:**

We aimed to analyze the association between hypertension and deep vein thrombosis (DVT) after orthopedic surgery.

**Methods:**

Relevant studies were identified by a search of PubMed, Embase, China National Knowledge Infrastructure, Wanfang, the Chinese Biomedical Literature, and Weipu database until December 2015. The association between hypertension and DVT after orthopedic surgery was assessed by pooled odds ratios (ORs) and 95 % confidence intervals (CIs). Heterogeneity was evaluated by the Chi-square test based on *Q* statistic and *I*^2^ statistics. Finally, publication bias was evaluated by Egger’s test.

**Results:**

A total of 16 articles with 68,955 males and 53,057 females were eventually identified. Studies yielded effects for homogeneous (*Q* = 38.41, *P* = 0.0008, and *I*^2^ = 60.9 %). Meta-analysis showed that hypertension was associated with DVT orthopedic surgery (OR 2.89, 95 % CI 2.18–3.83, *Z* = 7.38, *P* < 0.05). No statistical evidence of publication bias was found among studies (*t* = 1.90, *P* = 0.08). The funnel plot was symmetry, and the results were reliable.

**Conclusions:**

Hypertension may promote DVT after orthopedic surgery, and may be an important risk factor of DVT occurrence.

## Background

Hypertension is one of the major causes of disease burden all over the world [1]. In 2000, it was estimated that approximately 1 billion cases suffered hypertension and by 2025, the number is predicted to increase to 1.56 billion [2]. It is one of the most important risk factors for heart disease, stroke, coronary artery disease, and premature death [3]. Obesity, smoking, alcohol consumption, age, and education have been reported to play important roles in the risk of untreated and uncontrolled hypertension [4–6].

Deep vein thrombosis (DVT) is a systemic disease with a incidence of 67 per 100,000 of cases every year [7]. DVT could lead to postphlebitic syndrome, pulmonary embolism, and even death. In spite of adequate treatment, 1–8 % of patients developing pulmonary embolization will die [8, 9] and others will undergo long-term complications including chronic thromboembolic pulmonary hypertension and postphlebitic syndrome [10]. DVT is commonly associated with several co-morbidities. Over the past several years, studies on the association between DVT and hypertension have been reported, but the results are inconsistent. Some studies verified that hypertension could increase the development of DVT [11, 12]. However, Wang et al. [13] and Song et al. [14] reported that there was no statistically significant correlation between DVT and hypertension. Therefore, the controversial issue remains to be investigated.

Thus, in the current study, we performed a meta-analysis of available eligible studies to better elucidate the association between hypertension and DVT after orthopedic surgery.

## Methods

The paper did not involve any human or animal study, so the ethical approval was not required.

### Literature search

We searched electronic databases PubMed (http://www.ncbi.nlm.nih.gov/pubmed), Embase (http://www.embase.com), China National Knowledge Infrastructure (CNKI, http://www.cnki.net/), Wanfang (http://g.wanfangdata.com.cn/), the Chinese BioMedical Literature (CBM, http://www.sinomed.ac.cn/), and Weipu database (http://www.cqvip.com/) updated to December 2015 for all the publications on the association between hypertension and DVT. The search terms were hypertension or high blood pressure or HBP; deep vein thrombosis or thrombose veineuse profonde or DVT or deep venous thrombosis; orthopedic post-operation or orthopedic or orthopaedic and postoperative. Language restrictions were not used for the search.

### Study selection

Studies were included if they met the following criteria: (1) the observation group was patients with DVT after orthopedic surgery and the control group was patients without DVT after orthopedic surgery; (2) published on association between hypertension (blood pressure > 140/90 mm Hg) and DVT after orthopedic surgery in Chinese or English; (3) the number of patients with hypertension in the observation group and control group could be obtained. Studies were excluded if they were reviews, reports, or letters.

### Data extraction

With the standard protocol, two investigators independently extracted the following data from the included studies: the first author, publication year, study time and region, the number of patients in control group or observation group, the number of patients with hypertension, and the demographic characteristics (sex, age, hyperlipidemia, and diabetes mellitus). Disagreements were resolved through discussion or settled by a third reviewer.

### Statistical analysis

Meta-analysis was carried out using R 3.12 software. The odds ratio (OR) and its 95 % confidence interval (CI) were calculated for effect index. Heterogeneity test was evaluated by Chi-square based on *Q* statistic [15] and *I*^2^ statistics [16]. A random effects model was used to combine the data for the heterogeneous outcomes (*P* < 0.05 or *I*^2^ ≥ 50 %); otherwise, a fixed effects model was used [17]. A sensitivity analysis was performed, in which one study was removed at a time and others were analyzed to examine the influence of a single study on the combined OR value [18]. Publication bias was evaluated through funnel plot visual analysis with the Egger’s tests [19, 20]. A *P* value less than 0.05 was considered statistically significant.

## Results

### Characteristics of included studies

**Fig. 1:**
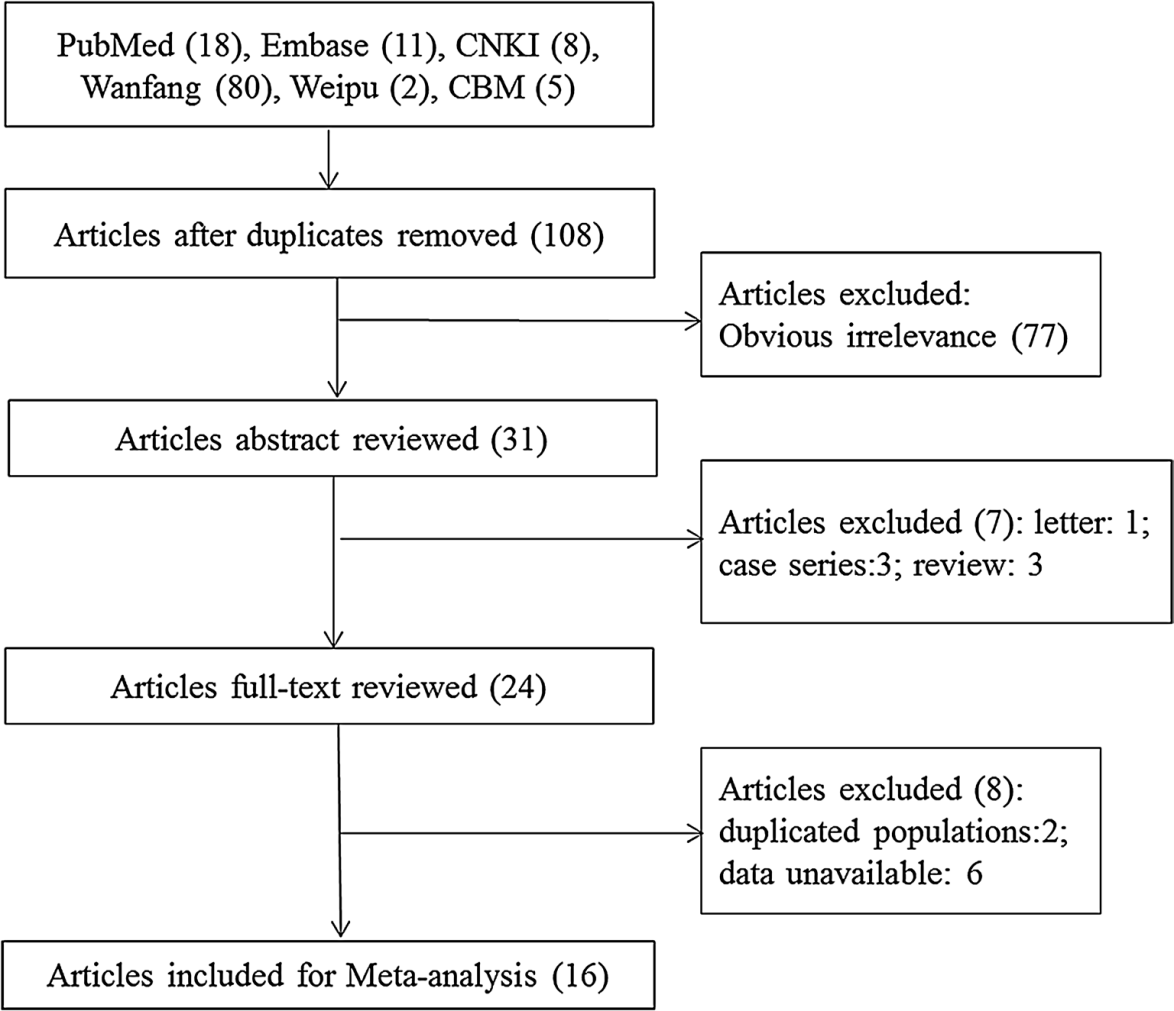
Flow diagram of study selection

The process of study selection was shown in Fig. 1. Initially, a total of 124 potentially relevant articles were retrieved from the databases (PubMed 18; Embase 11; CNKI 8; Wanfang 80; Weipu 2; CBM 5). Then, 108 articles were left after eliminating the duplicate publication, and 77 of them were excluded after screening the title and abstract. As a consequence, 31 articles were left and 15 (3 review, 1 letter, 3 case-report, 2 repeated people, and 6 did not provide sufficient data) of them were excluded after screening the full text. Finally, 16 articles [11, 12, 21–34] including 68,955 males and 53,057 females were included in this meta-analysis (Table 1). These studies were published between 2009 and 2015 with researches done between 2005 and 2014.

**Table 1:** Characteristics of included studies in the meta-analysis

Author	Public year	Study year	Study location	DVT	No.	Sex (M/F)	Age	Hyperlipidemia	Diabetes mellitus	Hypertension
Ma Jun	2009	2007.2–2007.7	Sichuan	Yes	17	4/13	15 (≥65)	15^a^	4	6
				No	34	9/25	16 (≥65)	7^a^	5	5
Zhang Ke-yun	2014	2010.2–2012.1	Hunan	Yes	28	6/22	28 (≥65)	24^a^	6	8
				No	64	17/47	30(≥65)	17^a^	10	12
Wu Fang-li	2011	2008.5–2009.12	Zhejiang	Yes	15	6/9	70.08 ± 12.18	NA	NA	9
				No	171	36/135	63.83 ± 10.6	NA	NA	36
Zheng Gui-juan	2015	2013.1–2014.6	Beijing	Yes	16	3/13	14 (≥65)	14^a^	6	5
				No	36	12/24	16 (≥65)	9^a^	4	7
He Han-liang	2014	2011.1–2013.12	Zhejiang	Yes	203	134/69	57 (>60)	73	NA	115
				No	3967	2399/1771	833 (>60)	1019	NA	1940
Yao Jie	2013	NA	Ningxia	Yes	212	145/67	76 (>64)	113^a^	20	43
				No	4921	3012/1909	854 (>64)	1113^a^	152	286
Rong Jin-yang	2013	2009.1–2013.4	Shanxi	Yes	130	76/54	48 (28–70)	30	33	48
				No	134	78/56	50 (30–72)	9	10	13
Long Jiang	2013	2009.5–2012.5	Yunnan	Yes	73	NA	49.3 ± 26.1	47	53	49
				No	72	NA		23	37	25
Ya Jun	2014	2010.3–2013.3	Yunnan	Yes	25	34/16	62.5 ± 1.2	NA	8	9
				No	25			NA	6	4
Sun Yong-fei	2011	2005.5–2010.10	Zhejiang	Yes	70	30/40	45.5 ± 7.1	NA	18	20
				No	70	30/40	45.6 ± 7.2	NA	8	9
Wang Da-wei	2012	2007.4–2011.4	Liaoning	Yes	91	101/64	38.2 ± 8.23	29	19	23
				No	74			6	6	8
Wang Xiao-feng	2013	2011.10–2012.11	Guangdong	Yes	52	64/39	59.8 ± 4.3	36	34	31
				No	51		39.4 ± 3.9	15	19	14
Huang Kun	2014	2010–2013	Jiangsu	Yes	80	57/23	51.3 ± 11.4	26	23	26
				No	80	55/25	50.8 ± 10.2	9	14	12
Guo Chang-jun	2013	2011.1–2013.1	Zhejiang	Yes	98	113/67	37.5 ± 1.2	22	28	22
				No	82			9	10	8
Yang Si-dong	2015	2013.7–2014.7	Hebei	Yes	147	410/451	54 (15–87)	NA	18	54
				No	714			NA	66	161
Zheng Sui-zhu	2013	2011.10–2012.10	Zhejiang	Yes	58	30/28	68.7 ± 17.1	NA	36	25
				No	62	32/30	55.6 ± 11.2	NA	8	10

^a^Triglyceride ≥ 1.7 mmol/L, *M* male, *F* female, *DVT* deep vein thrombosis, *NA* not available

### Merging quantitative data

**Fig. 2:**
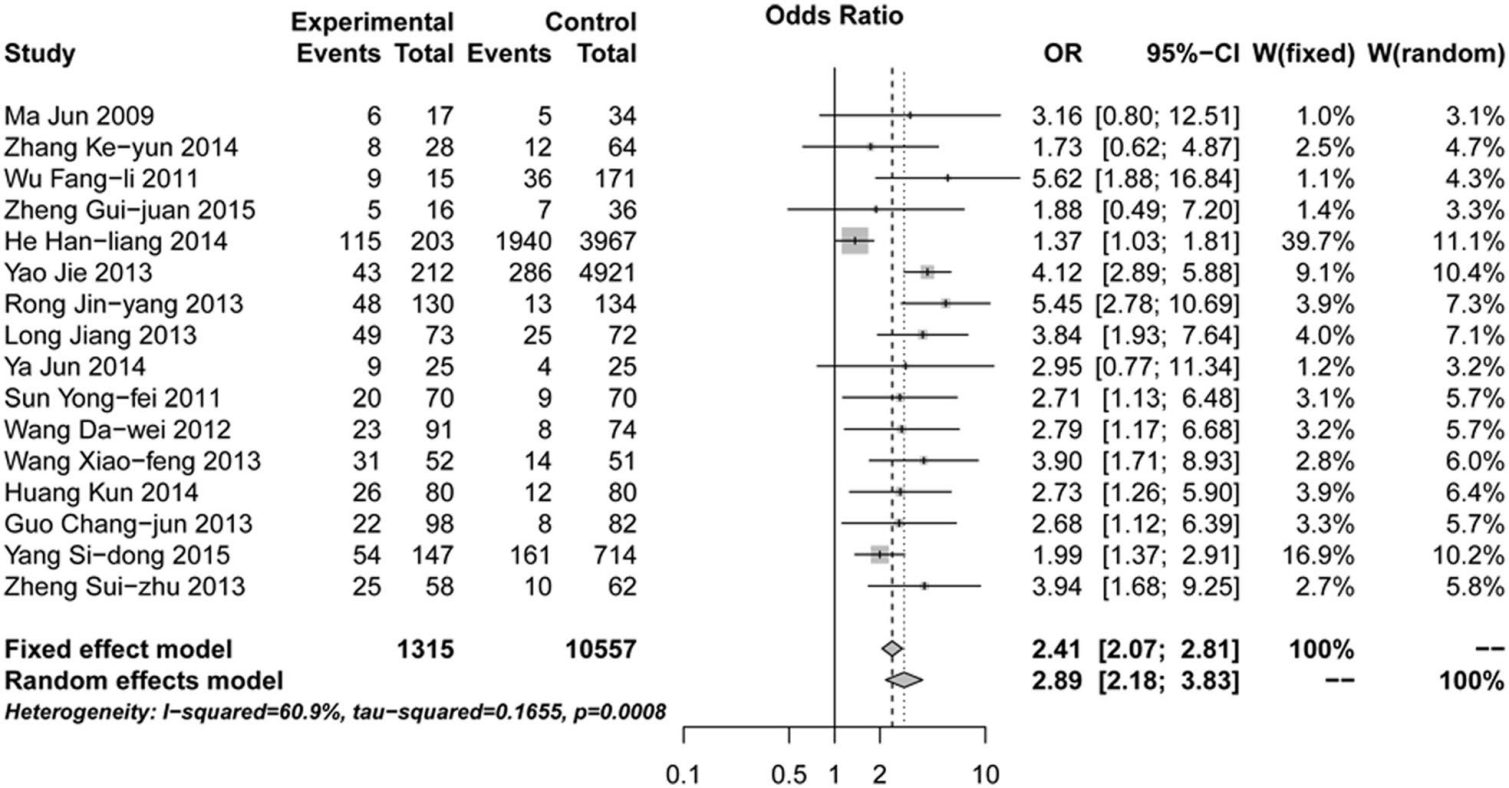
*Forest plot* of association between hypertension and deep vein thrombosis (DVT) after orthopedic surgery

**Fig. 3:**
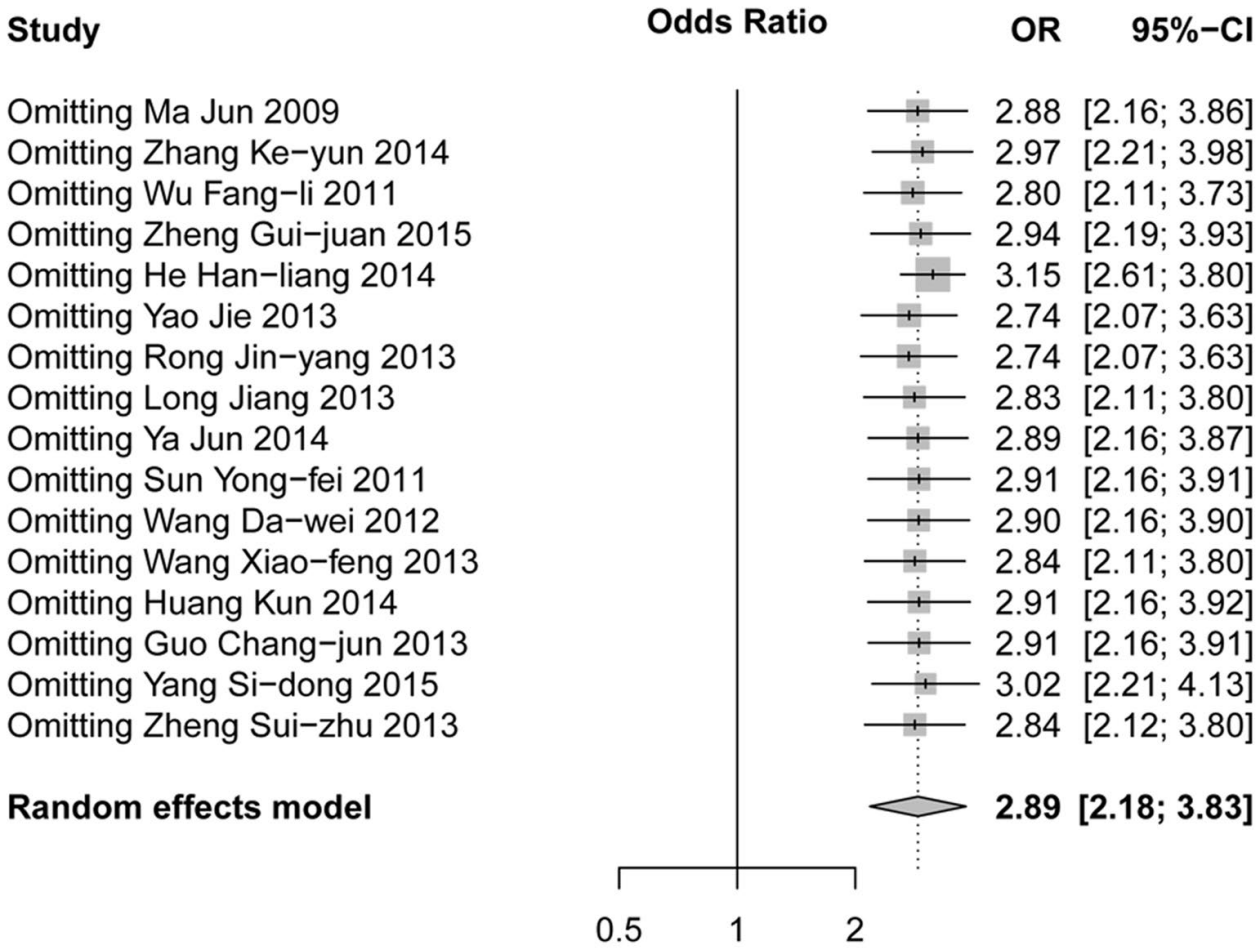
*Forest plot* for sensitivity analysis of association between hypertension and DVT after orthopedic surgery

**Fig. 4:**
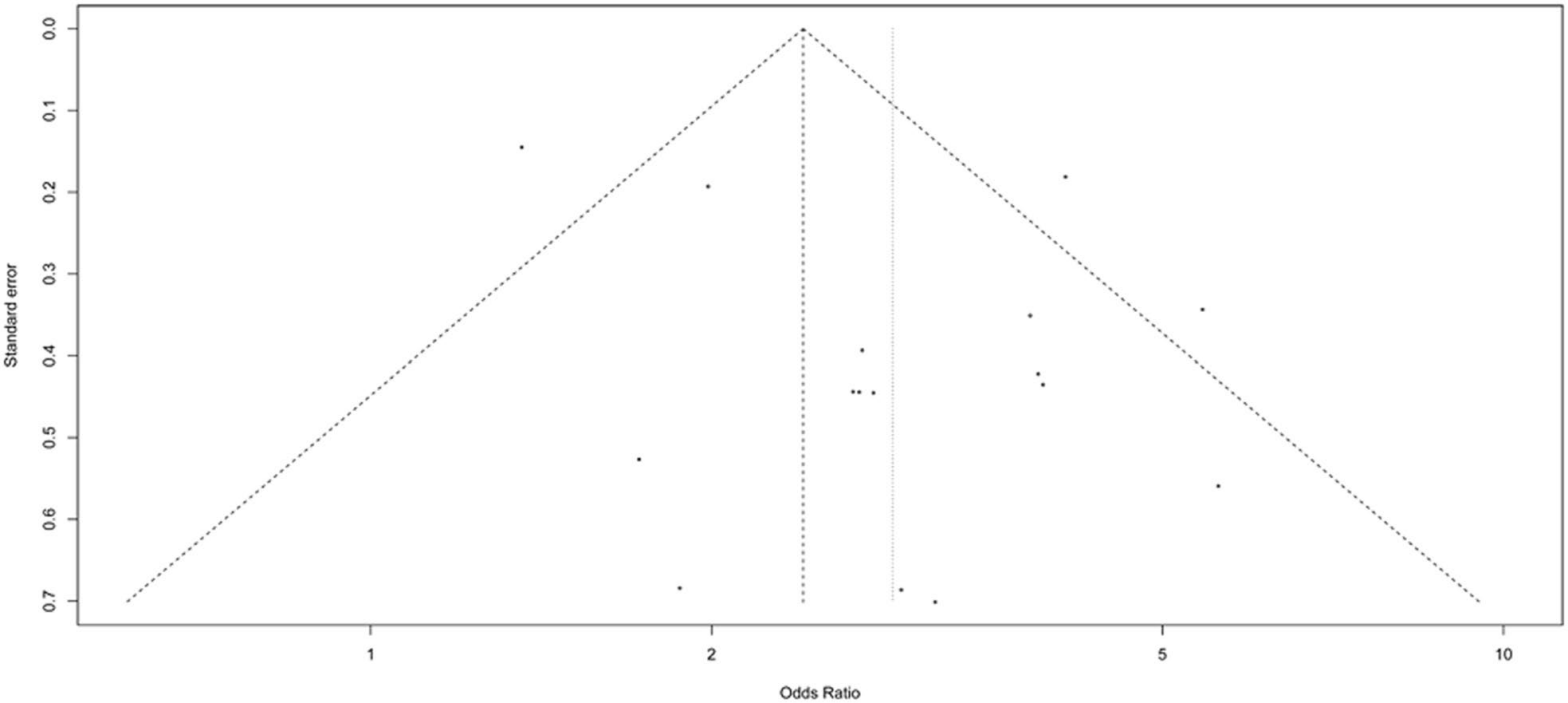
*Funnel plot* of association between hypertension and DVT after orthopedic surgery

The homogeneity analysis exhibited good with heterogeneity test (*Q* = 38.41, *P* = 0.0008, and *I*^2^ = 60.9 %). Then, the random effects model was used for further analysis. Meta-analysis showed that hypertension was associated with DVT after orthopedic surgery (OR 2.89, 95 % CI 2.18–3.83, Z 7.38, *P* < 0.05, Fig. 2). Sensitivity analysis showed that our results were stable (OR 2.89, 95 % CI: 2.18-3.83, Fig. 3). After Egger’s regression test, no publication bias among studies was found (t = 1.90, *P* = 0.08). The funnel plot was symmetry, so there was no publication bias and the result was reliable (Fig. 4).

### Subgroup analysis

Subgroup analysis pointed out that heterogeneity was decreased to different degrees (Table 2). In addition, the results of meta-analysis in each subgroup showed that hypertension may promote the formation of DVT after orthopedic surgery.

**Table 2:** Subgroup analyses of associations between hypertension and deep vein thrombosis after orthopedics surgery

Classification	Item	*N*	*I* ^2^ (%)	*P*	Model	OR [95 % CI]
Regional distribution	Western China	4	0	0.9498	*F*	3.89 [2.85–5.30]
	Central China	3	71.5	0.0298	*R*	2.70 [1.32–5.52]
	Eastern China	9	53.9	0.0268	*R*	3.95 [2.92–5.33]
The proportion of including	*N* < 500	13	0	0.8881	*F*	3.37 [2.63–4.31]
	*N* ≥ 500	3	91.5	<0.0001	*R*	2.23 [1.14–4.34]

*OR* odds ratio, *R* random effects model, *F* fixed effect model

## Discussion

This is the first systematic review and meta-analysis of studies, to our knowledge, examining the correlation between hypertension and DVT after orthopedic surgery. Totally 16 articles with 68,955 males and 53,057 females were included in this meta-analysis. The results showed that hypertension might promote DVT after orthopedic surgery (OR 2.89, 95 % CI 2.18–3.83, *Z* = 7.38).

In spite of the inherent risk of developing DVT for patients with orthopedic surgery, researches on risks of developing DVT were limited. Red blood cell storage has been found to be associated with increased incidence of DVT [35]. A previous report has indicated that patients with increased concentrations of factor VIII and von Willebrand’s factor have increased risk of DVT [36]. Compared with healthy controls, levels of red cell distribution width were higher in pre-hypertensive and hypertensive patients independently of age, inflammatory status, and anemia, suggesting the correlations between red cell distribution width and hypertension [37]. All of these may hint a potential relationship between DVT and hypertension.

The only risk factor of DVT in accordance with the conclusion from this meta-analysis is hypertension, which has already been verified previously. Several prospective studies have addressed the associations between hypertension and DVT. Patients with hypertension have been found with 2-fold increased likelihood of developing DVT [38]. In the current study, the results of meta-analysis in each subgroup have showed that hypertension may promote the formation of DVT after orthopedic surgery. In addition, hypertension has been found as an independent predictor of venous thromboembolism (VTE) in the general population [39]. In another study, after a prospective registry of 5451 patients with DVT, Goldhaber et al. [40] have found that 50 % patients have co-morbidities with hypertension. Kaisorn et al. [41] have also reported that hypertension may independently increase the risk of developing operative DVT (OR 1.785; 95 % CI 1.180–2.699; *P* = 0.006).

Age is a high prevalence of asymptomatic DVT event which has been identified in patients over 80 years [42]. A previous study with 102 consecutive patients of follow-up found that age greater than 65 years, body mass index (BMI) > 30 kg/m^2^, and smoking were risk factors for DVT [43]. In a recent study comprising 87 574 individuals found that obesity was a causal risk factor for DVT [44]. Examining on VTE, Chamberlain et al. [45] found that low-density lipoprotein cholesterol was not an risk factor of VTE. Another study with 855 men (65 VTE events) identified that smoking and waist circumference were risk factors for VTE, whereas high cholesterol and hypertension were not [46]. In addition, a Copenhagen City Heart Study pointed out that hypertension, smoking, and obesity were important risk factors for VTE, whereas total/high-density lipoprotein/low-density lipoprotein cholesterol, triglyceride, and diabetes mellitus were not [47].

Some limitations of this study should be addressed. First, only published studies were included and publication bias might exist, although no significant bias was detected by Egger’s test. Second, significant heterogeneity across studies was presented in overall and subgroup analysis, which might influence the pooled results. Third, although there was no limitation for language, only Chinese population was included, which might lead to bias. Fourth, as limited researches included in this meta-analysis, association between DVT and age, BMI, or gender was not analyzed. Finally, the small sample size was still insufficient to obtain a conclusive result. However, larger and well-designed studies based on different populations are warranted to validate our results.

In conclusion, this study showed that hypertension might promote DVT after orthopedic surgery, and it might be an important risk factor of DVT occurrence.
